# Enhanced Ionic Accessibility of Flexible MXene Electrodes Produced by Natural Sedimentation

**DOI:** 10.1007/s40820-020-00426-0

**Published:** 2020-04-11

**Authors:** Ning Sun, Zhaoruxin Guan, Qizhen Zhu, Babak Anasori, Yury Gogotsi, Bin Xu

**Affiliations:** 1 State Key Laboratory of Organic-Inorganic Composites, Beijing Key Laboratory of Electrochemical Process and Technology for Materials, Beijing University of Chemical Technology, Beijing, 100029 People’s Republic of China; 2 Department of Materials Science and Engineering and A. J. Drexel Nanomaterials Institute, Drexel University, Philadelphia, PA 19104 USA; 3 Department of Mechanical and Energy Engineering, Integrated Nanosystems Development Institute, Purdue School of Engineering and Technology, Indiana University – Purdue University Indianapolis, Indianapolis, IN 46202 USA

**Keywords:** MXene, Natural sedimentation, Vacuum filtration, Interlayer spacing, Li-storage

## Abstract

MXene nanosheets have been used for preparing highly flexible integrated electrodes due to their two-dimensional (2D) morphology, flexibility, high conductivity, and abundant functional groups. However, restacking of 2D nanosheets inhibits the ion transport in MXene electrodes, limiting their thickness, rate performance, and energy storage capacity. Here, we employed a natural sedimentation method instead of the conventional vacuum-assisted filtration to prepare flexible Ti_3_C_2_T_*x*_ MXene films with enlarged interlayer spacing, which facilitates the access of the lithium ions to the interlayers and thus leads to a greatly enhanced electrochemical performance. The naturally sedimented flexible film shows a double lithium storage capacity compared to the conventional vacuum-filtered MXene film, along with improved rate performance and excellent cycle stability.

**Electronic supplementary material:**

The online version of this article (10.1007/s40820-020-00426-0) contains supplementary material, which is available to authorized users.

## Highlights

- A simple, but effective strategy is proposed to prepare Ti_3_C_2_T_*x*_ MXene films by natural sedimentation method.
- The enlarged interlayer spacing of the prepared films facilitates the accessibility of the lithium ions between the interlayers and thus leads to a greatly enhanced electrochemical performance.
- The naturally sedimented MXene film shows a double lithium storage capacity compared to the conventional vacuum-filtered MXene film, along with improved rate performance and excellent cycle stability.

**Electronic supplementary material:**

The online version of this article (10.1007/s40820-020-00426-0) contains supplementary material, which is available to authorized users.

## Graphical abstract

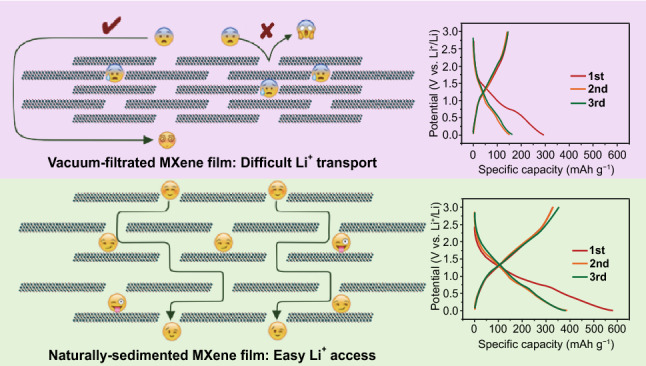

## Introduction

Currently, lithium-ion batteries (LIBs) dominate the portable electronics and electric vehicles markets due to their high energy and power density. However, with the ever-increasing attention on wearable electronic equipment, the conventional rigid LIBs cannot satisfy the flexibility requirements [1, 2]. The traditional method to prepare LIB electrodes is to coat a slurry consisting of active material, conductive agent and polymer binder onto metallic current collectors. When conventional LIBs are used in wearable and flexible equipment, the metallic current collectors not only add thickness to the device, but also might be easily deformed, causing the electrode material to detach from the current collector during the bending process, deteriorating the electrochemical performance. Therefore, manufacturing electrodes with excellent mechanical flexibility is one of the key challenges for fabricating flexible LIBs [3, 4]. Various materials, such as graphene, carbon nanotubes, and polymers, have been used in freestanding flexible electrodes, as well as electrodes placed on paper or fabrics [5–7]. However, the electrochemical properties of those materials need further improvement.

Transition metal carbides, nitrides and carbonitrides, a large family of two-dimensional (2D) materials known as MXenes, have been gaining a lot of interest in a variety of applications, especially for energy storage and conversion [8–10]. The characteristics of MXenes, including their unique 2D morphologies, rich chemistries, ultra-high electronic conductivities, and abundant surface functional groups, make them promising candidates for electrodes of supercapacitors (SCs) and LIBs [11–15]. Moreover, the excellent flexibility of MXene nanosheets endows their use for flexible electrode manufacturing. Via electrophoretic deposition, self-standing PPy/MXene flexible films with good electrochemical performance were prepared [16]. Using MXene as a flexible, electrochemically active and conductive binder, Yu et al. [17] prepared freestanding, flexible, MXene-bonded activated carbon film electrodes which demonstrated enhanced electrochemical performance when compared to conventional PVDF-bonded electrodes.

Pure MXene films perform well in SCs, which have high volumetric capacitance up to 1500 F cm^−3^ in acidic electrolyte [18], but the situation is different for LIBs. Previous reports based on density functional theory (DFT) computations predicted that Ti_3_C_2_T_*x*_, the most studied member of the MXene family, could be the host for lithium storage with a theoretical capacity up to 320 mAh g^−1^ based on the following reaction (Eq. 1) [19]:

1 $${\text{Ti}}_{3} {\text{C}}_{2} \, + \,2\,{\text{Li}}\, = \,{\text{Ti}}_{3} \,{\text{C}}_{2} \,{\text{Li}}_{2}$$

This value is comparable with the capacity of graphite (372 mAh g^−1^), the commercially used anode of LIBs. However, similar to other 2D materials, the restacking phenomenon of Ti_3_C_2_T_*x*_ flakes during the film fabrication process may decrease the ion accessibility and hinder their effective utilization. Thus, the reversible capacity for pristine MXene film is only 100–200 mAh g^−1^ [20–22], far from the theoretical value. Meanwhile, as the restacked large Ti_3_C_2_T_*x*_ flakes seriously impede the ion diffusion by increasing the diffusion path, and lowering diffusion kinetics, which is unfavorable for the rate performance. Many efforts have been made to improve the electrochemical performance of Ti_3_C_2_T_*x*_, such as interlayer spacing modulation [23–25], surface modification [26–28], and architecture design [29–31]. However, there are few reports on the pure MXene films with good lithium storage performance and flexibility.

Here, we propose a very simple, but effective strategy to prepare freestanding, flexible Ti_3_C_2_T_*x*_ MXene films by using natural sedimentation for LIBs. Compared to the routine vacuum-filtered MXene film with the restacked structure, the obtained sedimented MXene films exhibit enlarged interlayer distances, facilitating the ionic accessibility and ion transport. Therefore, the reversible capacity of the films increases dramatically from 145 to 351 mAh g^−1^, close to the theoretical capacity of Ti_3_C_2_T_*x*_. Besides, owing to the enhanced ionic accessibility, the rate performance and cyclability are also improved for the naturally sedimented MXene films.

## Experimental

### Preparation and Characterization

The MXene nanosheets were synthesized following the previous reported methods [32]. In a typical experiment, 1 g of Ti_3_AlC_2_ powder is slowly added to the solution of 0.99 g LiF and 10 mL HCl (12 M) under continuous stirring. After etching at 35 °C for 24 h, the resulting suspension is then washed with deionized water several times until the pH is around 6. To further delaminate the sheets, sonicating treatment for 1 h under Ar flow was followed. MXene nanosheets were obtained by collecting the supernatant after centrifugating the mixture solution at 3500 rpm for 1 h. The concentration of the Ti_3_C_2_T_*x*_ sheets is calculated by filtering a known volume of solution on Celgard 3501 membrane and measuring the weight of the obtained MXene film after drying at 60 °C in a vacuum oven.

Totally, 10 mg MXene nanosheets in above MXene colloidal solutions were dispersed in deionized water to certain concentrations (0.5, 1, and 2 M). Then the solutions were rested in the filters through Celgard 3501 membranes with a diameter of 4 cm for natural sedimentation and the obtained films were marked as Nat-0.5, Nat-1, and Nat-2, respectively. For comparison, the film prepared by vacuum filtering of 0.5 M MXene solution was named as Vac-0.5.

The morphology of the obtained MXene films was characterized on Hitachi S4800 scanning electron microscope (SEM). To guarantee that the exposed cross section is clear and uniform for SEM imaging, the MXene film was cut quickly with a sharp blade on a flat surface. The powder X-ray diffraction (XRD) was performed on a Bruker D8 Advanced X-ray diffractometer with Cu Kα radiation (*λ* = 0.154 nm). A Renishaw 1000 Raman spectrometer (514 nm) was used to record the Raman spectra.

### Electrochemical Measurement

The films were cut into small rounds with diameter of 10 mm, and directly used as the working electrodes. The mass loading of MXene was ~ 0.8 mg cm^−2^. With the as-prepared film as the working electrode, lithium foil as the counter electrode, a Celgard 3500 microporous membrane as the separator, and 1 M LiPF_6_ in ethylene carbonate/diethyl carbonate (*V*/*V* = 1:1) as the electrolyte, coin-type half cells were assembled in an argon-filled glove box (with O_2_ and H_2_O level below 0.1 ppm). The half-cells were tested within a voltage range of 0.01–3.0 V versus Li^+^/Li on a Land BT2000 battery test system (Wuhan, China). The cyclic voltammetry (CV) measurements were conducted at different scan rates between 0.01 and 3.0 V on a CHI600E electrochemical workstation (Chenhua, China). The electrochemical impedance spectroscopy (EIS) measurements were conducted within a frequency range of 1000 kHz to 0.1 Hz with amplitude of 10 mV on a VSP electrochemical workstation (Bio-Logic, France).

## Results and Discussion

**Fig. 1:**
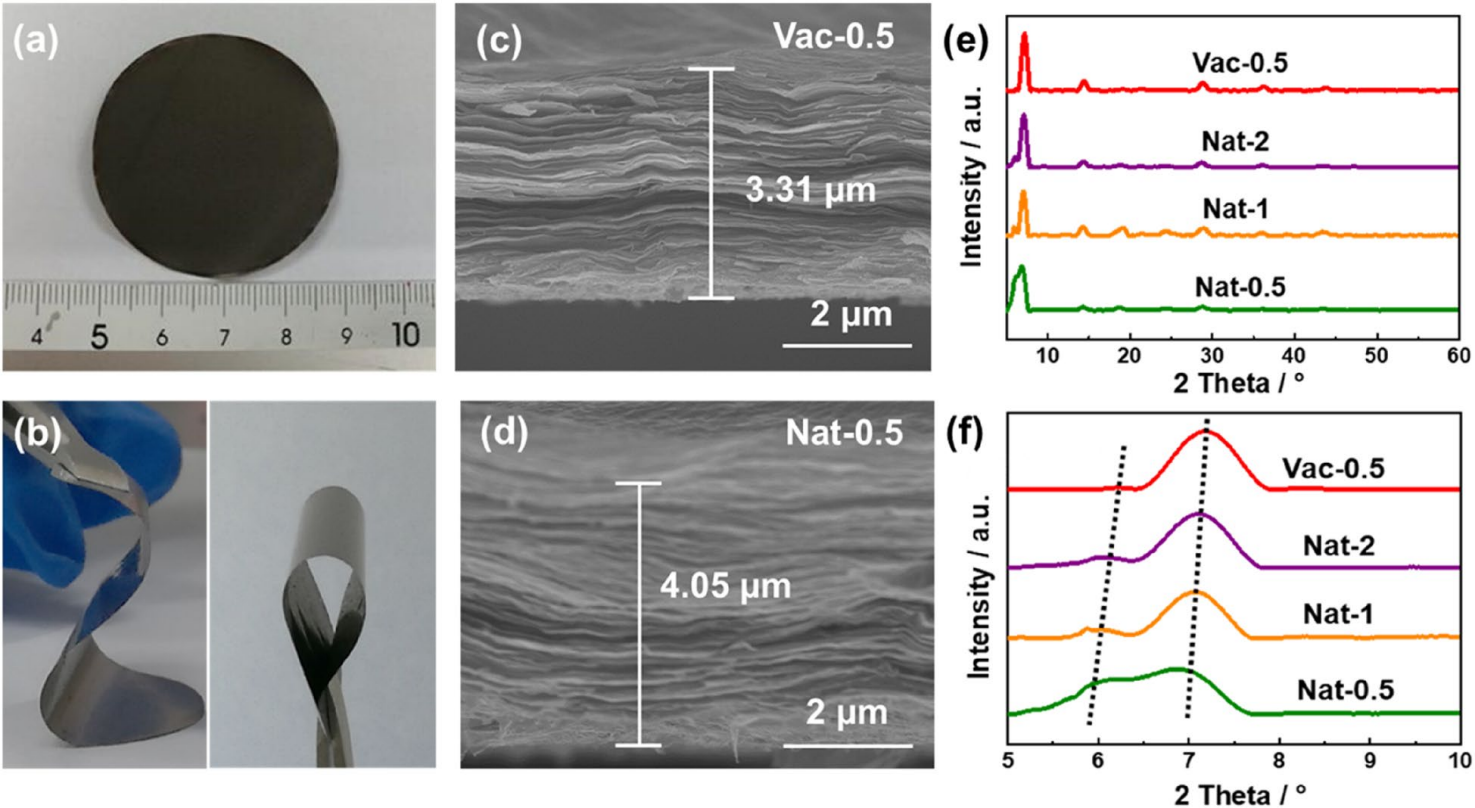
**a**, **b** Digital photographs of the flexible Nat-0.5 film. Cross-sectional SEM images of **c** Vac-0.5 film and **d** Nat-0.5 film. **e, f** XRD patterns of Vac-0.5, Nat-2, Nat-1, and Nat-0.5 films

The obtained freestanding MXene films demonstrated excellent flexibility, as shown in Fig. 1a, b, thus allowing for direct use as anodes with potential application in flexible or wearable energy storage devices. The cross-sectional SEM images (Fig. 1c, d) indicate that the vacuum-filtered and naturally sedimented MXene films show similar morphology with 2D delaminated MXene layers. However, unlike the vacuum-filtered film with a tightly stacked structure, the natural-sedimented films display a much more open structure. The thickness of Vac-0.5 film is only 3.31 μm, while Nat-0.5 film with the same mass shows a thickness of 4.05 μm (increase of 22%). This implies a dramatically enlarged interlayer distance for the naturally sedimented films, which can reduce the ionic diffusion barrier and improve the ionic accessibility between the MXene layers, providing a higher capacity and better rate performance. By adjusting the concentration of the MXene solutions, Nat-1 and Nat-2 also exhibit a thickness of 3.82 and 3.59 μm (Fig. S1), respectively, which are both larger than the vacuum-filtered film. The more dilute MXene solution leads to the thicker films with looser structures as more H_2_O molecules insert between the MXene layers, enabling larger interlayer spacing and allowing lithium ions to access the active sites [33].

XRD patterns show that all the naturally sedimented MXene films are similar to the vacuum-filtered film, without any impurity peaks, as shown in Fig. 1e. All the MXene films show a split (002) peak due to changing interlayer distances. However, the (002) peaks of the naturally sedimented films show a downshift tendency compared to Vac-0.5 film, indicating an expanded interlayer distance. According to the Bragg formula, the large and small interlayer distances were calculated as 14.06 and 12.20 Å for Vac-0.5, while their values increase to 14.76 and 12.56 Å for Nat-0.5 with a significant increase of 0.70 and 0.36 Å, respectively. Additionally, the intensity of the (002) peak at lower 2 theta increases in Nat-0.5, which is indicative of an increasing fraction of MXene flakes with a larger interlayer distance. For Nat-1 and Nat-2, the large/small interlayer distance values are 14.64/12.44 and 14.52/12.32 Å, respectively (Table S1). The enlarged interlayer distance contributes to the expanded thickness of the naturally sedimented MXene films and facilitates the ionic accessibility and diffusion in MXene films.

The Raman spectra of the as-prepared MXene films are displayed in Fig. S2. The vacuum-filtered MXene film shows typical Raman bands at 196 and 714 cm^−1^, corresponding to the A1g symmetry out-of-plane vibrations of Ti and C atoms, respectively. The modes at 287, 368 and 626 cm^−1^ relate to the Eg group vibrations, including in-plane (shear) modes of Ti, C, and surface functional group atoms [34, 35]. For the naturally sedimented MXene films, the absence of the peak at 144 cm^−1^ reveals that no TiO_2_ was generated during the longer sedimentation process. No obvious peak shift can be observed for the naturally sedimented MXene films, but the intensity of the out-of-plane vibrations becomes somewhat weaker in Nat-0.5.

**Fig. 2:**
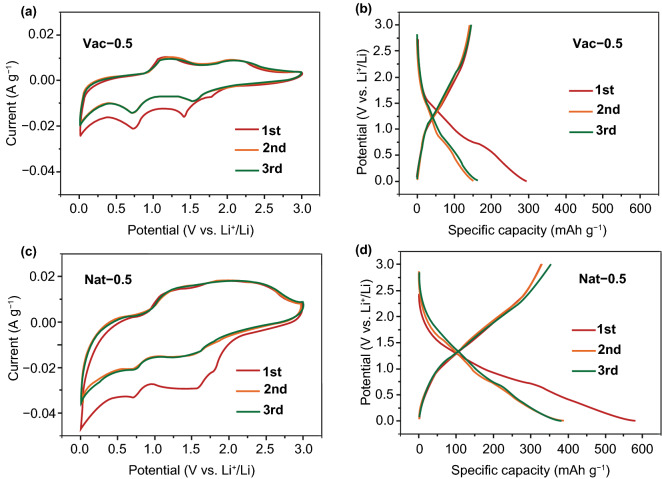
CV profiles at 0.1 mV s^−1^ and galvanostatic charge/discharge curves at 30 mA g^−1^ for the initial three cycles of **a**, **b** vacuum-filtered Vac-0.5 film and **c**, **d** naturally sedimented Nat-0.5 film

The freestanding, flexible MXene films were directly used as anodes for LIBs, and coin cells (CR2025) were fabricated using lithium foil as the counter electrode, and 1 M LiPF_6_ in ethylene carbonate/diethyl carbonate (*V*/*V* = 1:1) as the electrolyte. As shown in Fig. 2a, the cyclic voltammetry (CV) profile of the vacuum-filtered MXene film shows two reversible redox couples at 1.54/2.10 V and 0.71/1.09 V, corresponding to the lithium ions insertion/extraction between the MXene layers. However, the CV profile of Nat-0.5 exhibits a different electrochemical behavior as the two redox couples begin to merge (Fig. 2c). This phenomenon can be attributed to the enlarged interlayer distance which might cause the lithium storage mechanism in the MXene layers to change from a sequential to a simultaneous intercalation [31]. Nevertheless, the CV profiles for Nat-2 and Nat-1 (Fig. S3) have two reversible redox couples, as their interlayer distances are not large enough for the simultaneous intercalation behavior. Considering the much larger integral area of the CV profiles of Nat-0.5, the naturally sedimented films are expected to have a significantly higher reversible capacity than the vacuum-filtered ones.

The galvanostatic charge/discharge curves of all the prepared MXene films at 30 mA g^−1^ demonstrate a slope shape without any obvious plateaus. Similar to previous reported results [18–20], Vac-0.5 shows a limited reversible capacity of only 145 mAh g^−1^ with the initial Coulombic efficiency of 54.0%. The poor capacity results from the restacked MXene layers, which decreases the ion accessibility, impedes the ion diffusion and thus hinders their effective utilization. The initial irreversible capacity loss originates from the formation of solid electrolyte interface (SEI) and the possible side reaction of lithium ions with the abundant surface species on MXene. However, with an enlarged interlayer distance benefitting the ionic accessibility, Nat-0.5 exhibits a reversible capacity of 351 mAh g^−1^, more than twice that of Vac-0.5 and close to the theoretical capacity of Ti_3_C_2_. In addition, the initial Coulombic efficiency of Nat-0.5 is 56.5%, similar to that of Vac-0.5, which is a common value for MXene anode materials [20, 21] and indicates that the enlarged interlayer distance has little effect on the initial Coulombic efficiency. For Nat-2 and Nat-1, the reversible Li-storage capacities reach 191 and 328 mAh g^−1^ due to the expanded interlayer distance, showing a positive correlation between the capacity and the interlayer distance.

**Fig. 3:**
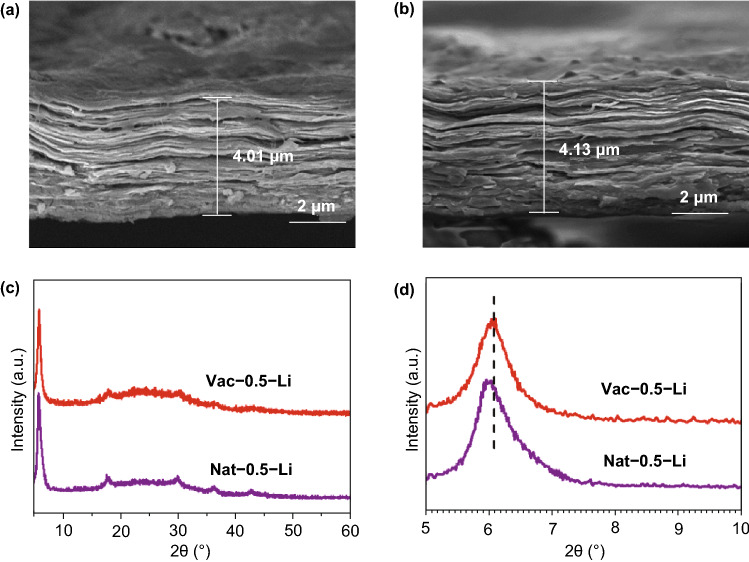
Cross-sectional SEM images of **a** Vac-0.5 and **b** Nat-0.5 after lithium ions insertion at the fifth cycle and **c**, **d** the corresponding XRD patterns

The lithium insertion between MXene layers is also confirmed by the cross-sectional SEM images (Fig. 3a, b) and XRD patterns of the discharged MXene films over 5 cycles (Fig. 3c, d). After discharged to 0.01 V, Vac-0.5 and Nat-0.5 films show similar morphology to the initial ones, but the thickness increases to 4.01 and 4.13 μm with a growth of 0.7 and 0.08 μm, respectively. Compared to the vacuum-filtered MXene film, the volume change for the naturally sedimented MXene films is negligible, implying a superb structural stability. XRD patterns show that the discharged Vac-0.5 and Nat-0.5 films display an interlayer distance of 14.59 and 14.92 Å, with an increase of 0.53 and 0.16 Å, respectively, compared to the interlayer distance of the pristine films before cycling. The natural-sedimented films with larger interlayer distance favor the insertion of lithium ions with less volume change, implying easier accessibility, and enhanced cycle stability and rate performance. Considering these change values are smaller than the size of solvent molecules, we infer that it is the desolvated lithium ions that are inserted/extracted into/from the stacked MXene layers during lithiation/delithiation, just as the case for sodium storage in MXene [36].

**Fig. 4:**
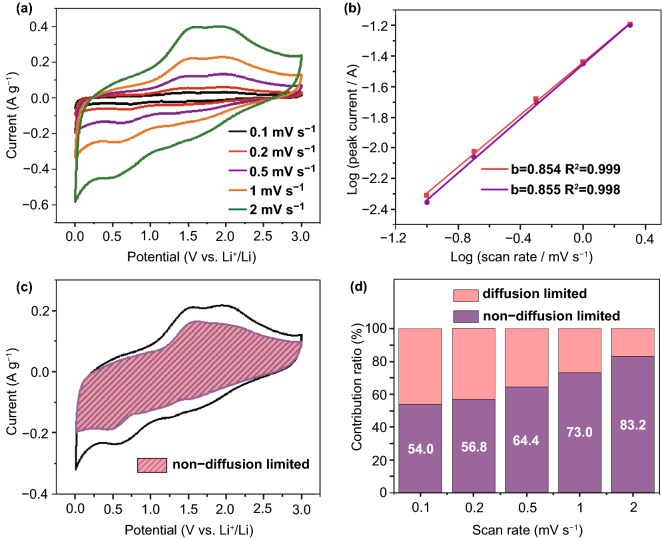
**a** CV curves at scan rates ranging from 0.1 to 2 mV s^−1^, **b** relationship between the peak current and scan rate based on the anodic peaks at ~ 2.0 (red) and ~ 1.5 V (purple), **c** CV profile collected at 1 mV s^−1^ with shaded area showing the contributions of the non-diffusion-limited processes, **d** survey of non-diffusion-limited current contributions at various scan rates of Nat-0.5 film. (Color figure online)

To further unveil the electrochemical kinetics for lithium storage in the naturally sedimented MXene films, CV curves at scan rates from 0.1 to 2 mV s^−1^ were recorded (Figs. 4a and S4). The mechanism of charge storage can be determined through the power-law relationship (Eq. 2):

2 $$i = \, av^{b}$$

where *i* is the measured current (A), *v* is the scan rate (V s^−1^), and *a* and *b* are fitting parameters [31, 37]. The fitting slope of the log(*ν*) − log(*i*) plots corresponds to the *b* value. A *b* value of 0.5 generally represents a diffusion-controlled intercalation, while a value of 1.0 indicates a surface-controlled process. As shown in Fig. S4d, the *b* value for the anodic peak at ~ 2.0 V of Vac-0.5 is 0.785, indicating that the charge storage in the vacuum-filtered MXene film is controlled by both the surface redox and diffusion-limited intercalation. However, the *b* value increases to 0.854 for Nat-0.5 (Fig. 4b), which implies less diffusional limitation for ionic transport in the naturally sedimented MXene film. The *b* values for Nat-1 and Nat-2 are 0.846 and 0.835, respectively, suggesting that the larger interlayer distance leads to larger contribution of surface processes in charge storage.

The non-diffusion-limited current at a certain scan rate can be determined by calculating the value of *k*_1_, according to Eq. 3:

3 $$i\left( V \right) \, = k_{1} \nu + k_{2} \nu^{1/2}$$

where *i* (V), *k*_1_*ν*, *k*_*2*_*ν*^1/2^, and *ν* represent the current (A) at a fixed potential, the non-diffusion-limited, and diffusion-controlled currents (A), and the scan rate, respectively [38, 39]. As shown in Fig. 4c, the shaded area represents the non-diffusion-limited contribution of Nat-0.5 at the scan rate of 1 mV s^−1^. Based on the quantitative analysis, the non-diffusion-limited current contributes 54.0% to the overall charge at 0.1 mV s^−1^, which gradually grows with the increasing scan rate and reaches 83.2% at 2 mV s^−1^ (Fig. 4d), indicating the fast kinetics for the non-diffusion-limited processes in the naturally sedimented MXene film.

**Fig. 5:**
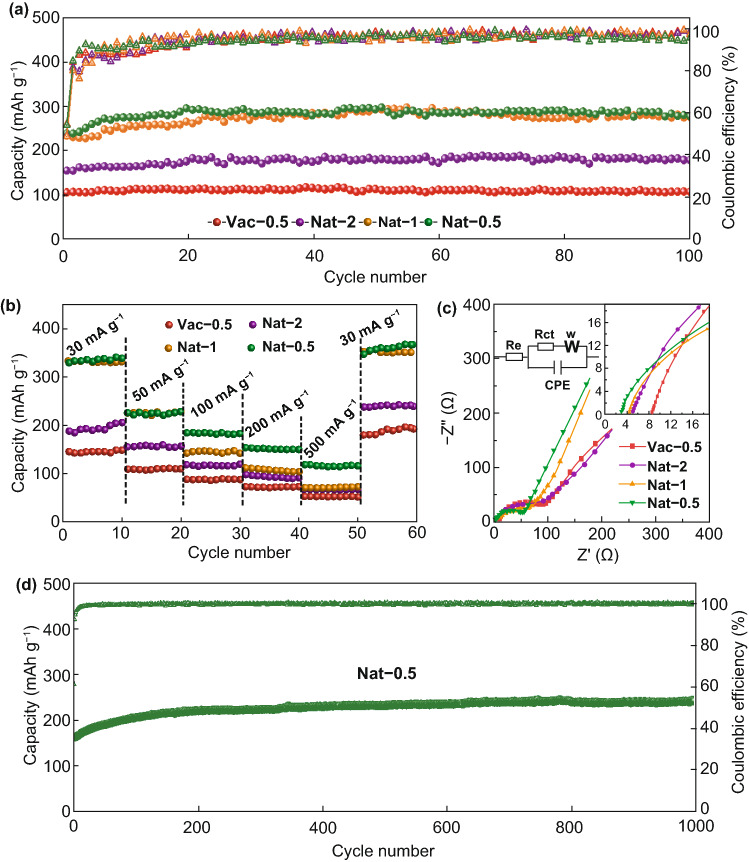
**a** Cycle performance at 50 mA g^−1^, **b** rate performance, and **c** Nyquist plots with the inset of the magnification of the high frequency area and the equivalent circuit of the vacuum-filtered and naturally sedimented MXene films. **d** Cycle performance of Nat-0.5 film at 200 mA g^−1^ for 1000 cycles

Figure 5a displays the cycle performance of all the prepared MXene films at 50 mA g^−1^. The naturally sedimented MXene films show much higher capacity than the routine vacuum-filtered film, together with good stability. After 100 cycles, the reversible capacity of Nat-0.5 maintains 266 mAh g^−1^ with no capacity fading, while Vac-0.5 only has a capacity of 104 mAh g^−1^. The outstanding cycle stability of the natural-sedimented MXene films is ascribed to the expanded interlayer distance, which can accommodate the volume change caused by the lithium ions insertion.

A comparison of the rate performances of the naturally sedimented (Fig. 5b) and the vacuum-filtered MXene films shows that Nat-0.5, benefitting from the enlarged interlayer distance, exhibits the highest capacity of 115 mAh g^−1^ at 500 mA g^−1^, more than two times larger than that of Vac-0.5 (53 mAh g^−1^). The larger interlayer distance promotes the ion transport between the MXene layers and allows fast charging. The cycle stability of Nat-0.5 film at the current density of 200 mA g^−1^ was tested, as shown in Fig. 5d. The reversible capacity of 242 mAh g^−1^ was maintained after 1000 cycles, with no capacity loss compared to the first cycle. The lithium storage performance of Nat-0.5 is further compared with other reported pure Ti_3_C_2_T_*x*_ MXene anodes, as shown in Table S2. It can be seen that the naturally sedimented MXene with enlarged interlayer distance and improved ionic accessibility shows very competitive Li-storage performance, indicating that natural sedimentation is a simple, but effective strategy to prepare high-performance flexible MXene electrodes.

The improved dynamic properties of the naturally sedimented MXene films can be explained by the electrochemical impedance spectroscopy (EIS) test (Fig. 5c) [40, 41]. Fitting with the equivalent circuit, the ohmic resistance (*R*_e_) and charge transfer resistance (*R*_ct_) of Nat-0.5 dramatically decrease to 3.1 and 49.7 Ω (Table S3), respectively, less than half of Vac-0.5 with *R*_e_ of 8.9 and *R*_ct_ of 105.2 Ω. Besides, the steeper sloping linear range in the low frequency corresponds to smaller ion diffusion resistance (*R*_Li_) in Nat-0.5, compared to Vac-0.5, indicating the faster ion diffusion and improved electrochemical kinetics.

**Fig. 6:**
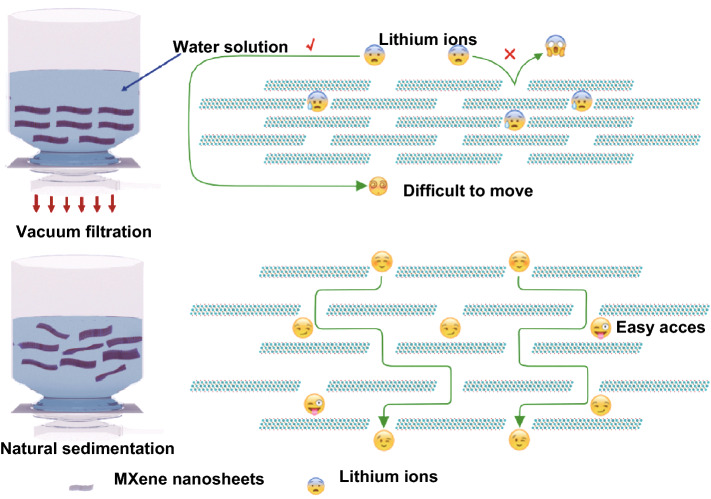
Comparison of improved ionic accessibility of the naturally sedimented MXene films and the conventional vacuum-filtered MXene film

Just as Fig. 6 shows, naturally sedimented strategy can effectively avoid the restacking phenomenon of 2D Ti_3_C_2_T_*x*_ flakes during the film fabrication process. The loose layer structure with enlarged interlayer distance can improve the ion accessibility, achieving the effective utilization the 2D Ti_3_C_2_T_*x*_ flakes. Meanwhile, the open structure is favorable for ion diffusion, leading to improved rate capability. Therefore, compared with the conventional vacuum-filtered Ti_3_C_2_T_*x*_ MXene film, the naturally sedimented Ti_3_C_2_T_*x*_ MXene films show greatly enhanced electrochemical performance, including high Li-storage capacity and excellent cycle stability and rate performance.

## Conclusions

In summary, we prepared freestanding, flexible Ti_3_C_2_T_*x*_ MXene films by simple natural sedimentation as anodes for LIBs. Without the vacuum forcing the sheets to stack and align within the film plane, the obtained naturally sedimented MXene films exhibit enlarged interlayer distance, which facilitates the ionic accessibility and fast ion transfer. Thus, the electrochemical lithium storage performance is significantly enhanced. The reversible capacity of the naturally sedimented MXene film is almost twice that of the vacuum-filtered MXene film and reaches 351 mAh g^−1^ at 30 mA g^−1^ with greatly improved rate performance and cycle stability. Combined with the very simple and easy-to-scale-up process, natural sedimentation is a promising strategy to prepare high-performance flexible anodes for LIBs and other types of batteries.

## Electronic supplementary material

Below is the link to the electronic supplementary material.

Supplementary material 1 (PDF 600 kb)
