# Supplementary material for: Enhanced Ionic Accessibility of Flexible MXene Electrodes Produced by Natural Sedimentation
Source: Nanomicro Lett. 2020 Apr 11;12:89. doi: 10.1007/s40820-020-00426-0 (PMC7770857; doi:10.1007/s40820-020-00426-0)
Supplement: Supplementary file 1 — Supplementary material 1 (PDF 600 kb) [file 40820_2020_426_MOESM1_ESM.pdf]

Supporting Information for

Enhanced Ionic Accessibility of Flexible MXene Electrodes Produced by Natural Sedimentation

Ning Sun[1, †], Zhaoruxin Guan[1, †], Qizhen Zhu[1], Babak Anasori[2, 3], Yury Gogotsi[2, *], Bin Xu[1, *]

[1]State Key Laboratory of Organic-Inorganic Composites, Beijing Key Laboratory of Electrochemical Process and Technology for Materials, Beijing University of Chemical Technology, Beijing 100029, People's Republic of China

[2]Department of Materials Science and Engineering and A. J. Drexel Nanomaterials Institute, Drexel University, Philadelphia, PA 19104, USA

[3]Integrated Nanosystems Development Institute, Department of Mechanical and Energy Engineering, Purdue School of Engineering and Technology, Indiana University – Purdue University Indianapolis, Indianapolis, IN 46202, USA

[†]Ning Sun and Zhaoruxin Guan contributed equally to this work

*Corresponding authors. E-mail: binxumail@163.com or xubin@mail.buct.edu.cn (B. Xu), gogotsi@drexel.edu (Y. Gogotsi)

Supplementary Tables and Figures

Table S1 2θ for the split (002) peaks and the calculated interlayer distances of the conventional vacuum-filtered MXene film and natural-sedimented MXene films

	high-angle peak		low-angle peak	
	2θ	d (Å)	2θ	d (Å)
**Vac-0.5**	7.24	12.20	6.28	14.06
**Nat-2**	7.17	12.32	6.08	14.52
**Nat-1**	7.10	12.44	6.03	14.64
**Nat-0.5**	7.03	12.56	5.98	14.76

Table S2 Comparison of lithium storage performance between naturally-sedimented MXene and other reported pure $Ti_3C_2T_x$ MXene anode materials

Electrode material	Li-storage capacity	Cycle performance	Rate performance	Refs.
**Free-standing $Ti_3C_2T_x$ MXene film prepared by natural sedimentation**	**351 mAh g^{-1} at 30 mA g^{-1}**	**242 mAh g^{-1} at 320 mA g^{-1} over 1000 cycles (no capacity loss)**	**115 mAh g^{-1} at 500 mA g^{-1}**	**This work**
Heteroatom-controlled $Ti_3C_2T_x$ MXene films by annealing	221 mAh g^{-1} at 32 mA g^{-1}	~100 mAh g^{-1} at 320 mA g^{-1} over 500 cycles	124 mAh g^{-1} at 320 mA g^{-1}	[S1]
Free-standing $Ti_3C_2T_x$ electrode prepared by cold pressed	120 mAh g^{-1} at 30 mA g^{-1} (electrode thickness: 220 μm)	28 mAh g^{-1} over 50 cycles		[S2]
Al^{3+} pre-intercalated $Ti_3C_2T_x$ film electrode	157.6 mAh g^{-1} at 1 C	Retaining 85% over 100 cycles	42.5 mAh g^{-1} at 5 C	[S3]
Low-F Ti_3C_2 MXene film prepared by annealing	~123.7 mAh cm^{-3} at 1 C	Retaining 75% over 100 cycles	~50 mAh cm^{-3} at 5 C	[S4]
$Ti_3C_2T_x$ MXene film treated with hydrazine vapor and annealing	~180 mAh g^{-1} at 100 mA g^{-1}	56.4 mAh g^{-1} at 1A g^{-1} over 1000 cycles	80 mAh g^{-1} at 1 A g^{-1}	[S5]
$Ti_3C_2T_x$ paper prepared by intercalation with hydrazine monohydrate	410 mAh g^{-1} at 1 C	—	—	[S6]
Porous $Ti_3C_2T_x$ film	~110 mAh g^1 at 0.5 C	Retaining ~100% over 100 cycles		[S7]
$Ti_3C_2T_x$/CNT composite films (9:1)	220 mAh g^1 at 0.5 C	Retaining ~100% over 100 cycles		[S7]
Porous $Ti_3C_2T_x$/CNT composite films (9:1)	650 mAh g^1 at 0.1 C	Capacity increases over 100 cycles	~230 mAh g^1 at 10 C	[S7]
Ti_3C_2/CNTs hybrid film (1:1)	403.5 mAh g^{-1} at 0.5 C	428.1 mAh g^{-1} over 300 cycles	218.2 mAh g^{-1} at 2 C	[S8]
Ti_3C_2 intercalated with DMSO	~210 mAh g^{-1} at 26 mA g^{-1}	118 mAh g^{-1} at 260 mA g^{-1} over 75 cycles	123.6 mAh g^{-1} at 260 mA g^{-1}	[S9]
Nitrogen containing Ti_3C_2 prepared by heat treatment in NH_3	~250 mAh g^{-1} at 32 mA g^{-1}		168 mAh g^{-1} at 320 mA g^{-1}	[S10]
Multilayer Ti_3C_2 MXene improved by calcination	254.6 mAh g^{-1} at 0.1 C	147.4 mAh g^{-1} at 1 C over 100 cycles	120 mAh g^{-1} at 4 C	[S11]

Table S3 The fitting resistance of the obtained MXene films

	Vac-0.5	Nat-2	Nat-1	Nat-0.5
**R_e (Ω)**	8.9	5.1	4.3	3.1
**R_{ct} (Ω)**	105.2	89.2	67.5	49.7
**R_{Li} (Ω)**	595.6	540.9	347.1	325.6

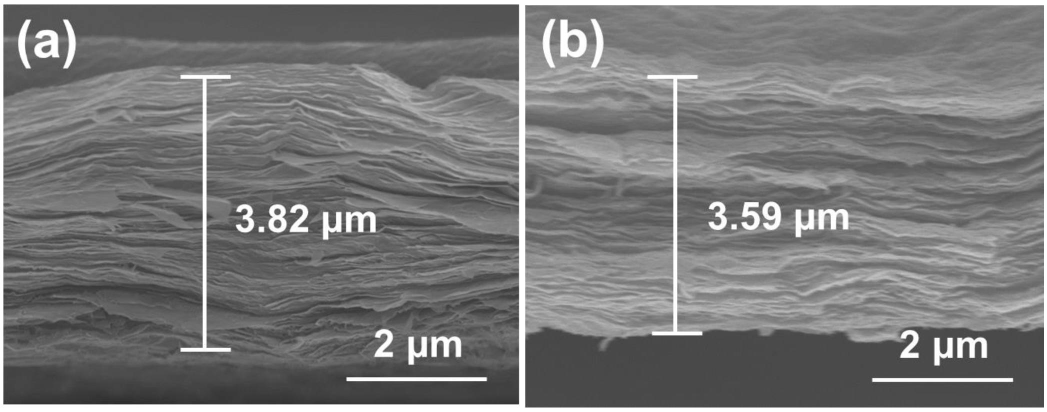

Fig. S1 Cross-sectional SEM images of Nat-1 film (**a**) and Nat-2 film (**b**)

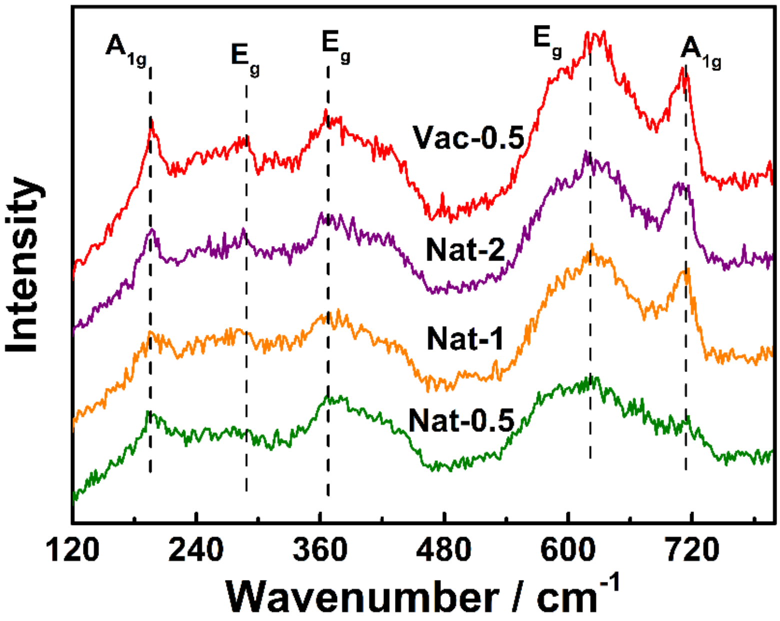

Fig. S2 Raman spectra of the as-prepared MXene films

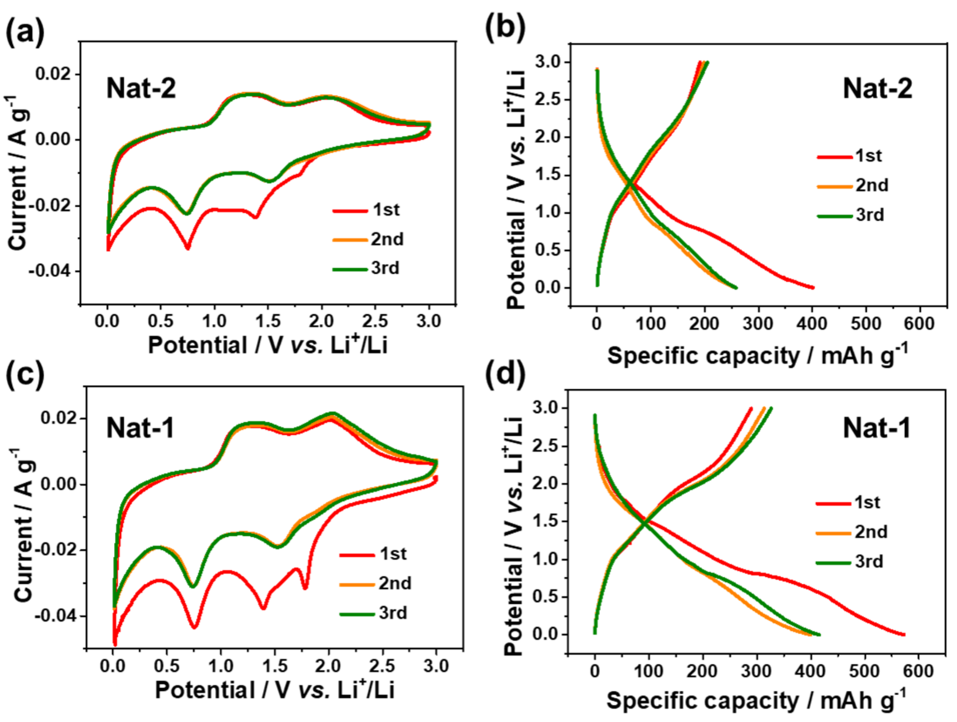

Fig. S3 CV profiles at 0.1 mV s^{-1} and galvanostatic charge/discharge curves at 30 mA g^{-1} for the initial three cycles of Nat-2 film (**a, b**) and Nat-1 film (**c, d**)

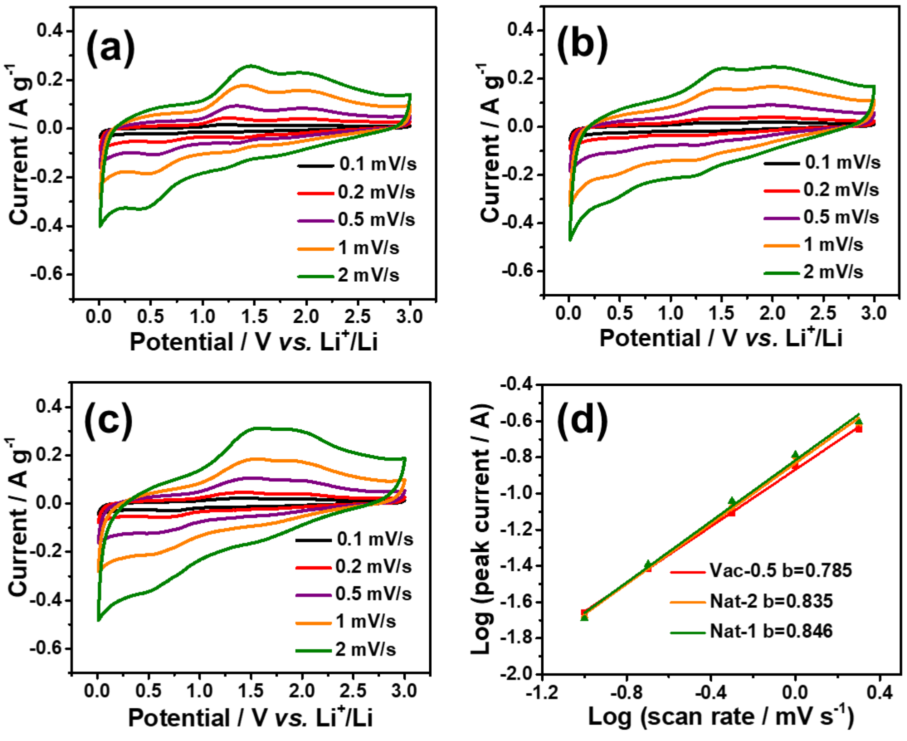

Fig. S4 CV curves at various scan rates ranging from 0.1 to 2 mV s^{-1} of Vac-0.5 (**a**), Nat-2 (**b**) and Nat-1 (**c**) and the relationships between the peak current and scan rate for the anodic peak at ~2.0 V of the prepared MXene films (**d**)

Supplementary References

[S1]Zhang H., Xin X., Liu H., Huang H., Chen N., et al., Enhancing lithium adsorption and diffusion toward extraordinary lithium storage capability of freestanding $Ti_3C_2T_x$ MXene. J. Phys. Chem. C **123**(5), 2792-2800 (2019). https://doi.org/10.1021/acs.jpcc.8b11255

[S2]Kim S.J., Naguib M., Zhao M.Q., Zhang C.F., Jung H.T., Barsoum M.W., Gogotsi Y., High mass loading, binder-free MXene anodes for high areal capacity Li-ion batteries. Electrochim. Acta **163**, 246-251 (2015). https://doi.org/10.1016/j.electacta.2015.02.132

[S3]Lu M., Han W., Li H., Shi W., Wang J., et al., Tent-pitching-inspired high-valence period 3-cation pre-intercalation excels for anode of 2D titanium carbide (MXene) with high Li storage capacity. Energy Storage Mater. **16,** 163-168 (2019). https://doi.org/10.1016/j.ensm.2018.04.029

[S4]Lu M., Li H., Han W., Chen J., Shi W., et al., 2D titanium carbide (MXene) electrodes with lower-F surface for high performance lithium-ion batteries. J. Energy. Chem. **31**, 148-153 (2019). https://doi.org/10.1016/j.jechem.2018.05.017

[S5]Ma Z., Zhou X., Deng W., Lei D., Liu Z., 3D porous MXene (Ti_3C_2)/reduced graphene oxide hybrid films for advanced lithium storage. ACS Appl. Mater. Interfaces **10**(4), 3634-3643 (2018). https://doi.org/10.1021/acsami.7b17386

[S6]Mashtalir O., Naguib M., Mochalin V.N., Dall'Agnese Y., Heon M., Barsoum M. W., Gogotsi Y., Intercalation and delamination of layered carbides and carbonitrides. Nat. Commun. **4**, 1716 (2013). https://doi.org/10.1038/ncomms2664

[S7]Ren C.E., Zhao M.Q., Makaryan T., Halim J., Boota M., et al., Porous two-dimensional transition metal carbide (MXene) flakes for high-performance Li-ion storage. Chemelectrochem **3**(5), 689-693 (2016). https://doi.org/10.1002/celc.201600059

[S8]Liu Y., Wang W., Ying Y., Wang Y., Peng X., Binder-free layered Ti_3C_2/CNTs nanocomposite anodes with enhanced capacity and long-cycle life for lithium-ion batteries. Dalton Trans. **44**(16), 7123-7126 (2015). https://doi.org/10.1039/c4dt02058h

[S9]Sun D., Wang M., Li Z., Fan G., Fan L.-Z., Zhou A., Two-dimensional Ti_3C_2 as anode material for Li-ion batteries. Electrochem. Commun. **47**, 80-83 (2014). https://doi.org/10.1016/j.elecom.2014.07.026

[S10] Cheng R., Hu T., Zhang H., Wang C., Hu M., et al., Understanding the lithium storage mechanism of $Ti_3C_2T_x$ mxene. J. Phys. Chem. C **123**(2), 1099-1109 (2018). https://doi.org/10.1021/acs.jpcc.8b10790

[S11] Kong F., He X., Liu Q., Qi X., Zheng Y., Wang R., Bai Y., Improving the electrochemical properties of MXene Ti_3C_2 multilayer for Li-ion batteries by vacuum calcination. Electrochim. Acta **265**, 140-150 (2018). https://doi.org/10.1016/j.electacta.2018.01.196
